# Expression of AMPA and NMDA receptor subunits in the cervical spinal cord of wobbler mice

**DOI:** 10.1186/1471-2202-7-71

**Published:** 2006-10-26

**Authors:** Paolo Bigini, Fabrizio Gardoni, Sara Barbera, Alfredo Cagnotto, Elena Fumagalli, Annalisa Longhi, Massimiliano M Corsi, Monica Di Luca, Tiziana Mennini

**Affiliations:** 1 Laboratory of Receptor Pharmacology, Department of Biochemistry and Molecular Pharmacology, Mario Negri Institute for Pharmacological Research, Milano, Italy; 2 Centre of Excellence on Neurodegenerative Diseases and Department of Pharmacological Sciences, University of Milan, 20133 Milano, Italy; 3 Institute of General Pathology, University of Milan, Milano, Italy

## Abstract

**Background:**

The localisation of AMPA and NMDA receptor subunits was studied in a model of degeneration of cervical spinal motoneurons, the wobbler mouse. Cervical regions from early or late symptomatic wobbler mice (4 or 12 weeks of age) were compared to lumbar tracts (unaffected) and to those of healthy mice.

**Results:**

No differences were found in the distribution of AMPA and NMDA receptor subunits at both ages. Western blots analysis showed a trend of reduction in AMPA and NMDA receptor subunits, mainly GluR1 and NR2A, exclusively in the cervical region of late symptomatic mice in the triton-insoluble post-synaptic fraction but not whole homogenates. Colocalisation experiments evidenced the expression of GluR1 and NR2A receptors in activated astrocytes from the cervical spinal cord of wobbler mice, GluR2 did not colocalise with GFAP positive cells. No differences were found in the expression of AMPA and NMDA receptor subunits in the lumbar tract of wobbler mice, where neither motoneuron loss nor reactive gliosis occurs.

**Conclusion:**

In late symptomatic wobbler mice altered levels of GluR1 and NR2A receptor subunits may be a consequence of motoneuron loss rather than an early feature of motoneuron vulnerability.

## Background

Amyotrophic lateral sclerosis (ALS) is a neurodegenerative disorder affecting motoneurons in the spinal cord, brainstem and motor cortex and leading to denervation, muscular atrophy, paralysis and premature death [1]. The disease is sporadic in approximately 90% of cases [2] and the correlation between the pathology and an identified gene mutation is known only in a small percentage of cases (2%) [3].

Glutamate-induced excitotoxicity may be one of the main factors in ALS pathogenesis [4]. Both glial and neuronal glutamate transporters play a pivotal role in avoiding excitotoxicity by removing the excess of glutamate released into the synaptic cleft from presynaptic neurons and consequently preventing the overstimulation of post-synaptic glutamate receptors. Evidence of abnormal glutamate metabolism and impaired expression of the glial glutamate transporter 2 (EAAT2) in ALS patients suggests that glutamate-induced excitotoxicity plays a key role in generating this disease [5]. Glutamate overstimulation can act through both the N-methyl-D-aspartate (NMDA) receptors and the alpha-amino-3-hydroxy-5-methyl-4-isoxazole propionate/kainate ionotropic (AMPA) receptors, generating an excessive influx of Ca^++ ^and Na^+ ^in neurons and the subsequent activation of damaging pathways, leading to motoneuron death [5]. Another source of motoneuron vulnerability involves a change of Ca^++ ^conductance in AMPA receptors. The relative Ca^++ ^permeability of native AMPA receptors in neurons is inversely correlated with the rate of edited GluR2 and the differences in this Ca^++ ^permeability between various neuronal cell types could be an important constituent of selective vulnerability [5]. It has been widely demonstrated that different neuronal cell types can differ in GluR2 expression, in the rate of GluR2 editing and in the desensitisation properties of their AMPA receptors [6] and such differences may be related to the selective vulnerability of motoneurons in ALS. GluR2 mRNA levels in motoneurons are the lowest among the human neuronal populations considered [7]. In addition, in a subset of ALS patients, the editing of GluR2 is defective, causing increased Ca^++ ^permeability to the AMPA receptor [8], that enhances Ca^++^-dependent pathways and leads to motoneurons death [9].

Although these results suggest glutamate-induced excitotoxicity is involved in ALS, it is almost impossible to verify this in humans because of the impossibility of studying cerebral tissues during the clinical course of the disease. Therefore animal models of motoneuron degeneration can provide a reliable tool for investigating alterations of parameters potentially involved in the human disease, mainly during the early phases.

The wobbler mouse, originally characterized and described by Falconer [10], carries a mutation in a gene coding for a protein involved in the retrograde transport from endosomes to the trans Golgi network (Vps54) [11]. At the end of the symptomatic phase (12 weeks of age) the number of motoneurons in the cervical region is reduced by about 65%. Wobbler mice show progressive atrophy of foreleg muscles, with marked loss of muscle strength and motor ability.

Although several pharmacological treatments have been tested in wobbler mice and a full ultrastructural characterization of degenerating motoneurons has already been carried out [12-16], the possible role of glutamate-induced excitotoxicity in motoneuron death in these mice is far from clear. No changes in the GLT-1 and GLAST glutamate transporters were reported in cervical spinal cord at different stages of disease [17] and results on glutamate receptor binding autoradiography in wobbler spinal cord tend to vary [18,19].

To better elucidate the role of glutamate receptors in this model of motoneuron disease, we focused on the expression and localisation of AMPA and NMDA receptor subunits. We performed Western blot experiments to evaluate the protein levels of the different AMPA (GluR1-4) and NMDA (NR1, NR2A) receptor subunits both in whole homogenates and in Triton-Insoluble post-synaptic Fraction (TIF) [20] from cervical and lumbar spinal cord. Since motoneurons represent only a small percentage of the heterogeneous tissue of spinal cord, we also investigated AMPA and NMDA receptor subunits by immunohistochemical experiments, to obtain information both on the levels of protein expression and on the cellular localization in motoneurons of cervical and lumbar spinal cord in healthy and diseased mice at two different phases of the disease.

## Results

### Immunohistochemistry for AMPA and NMDA receptors

#### Cervical region

##### GluR1

**Figure 1:**
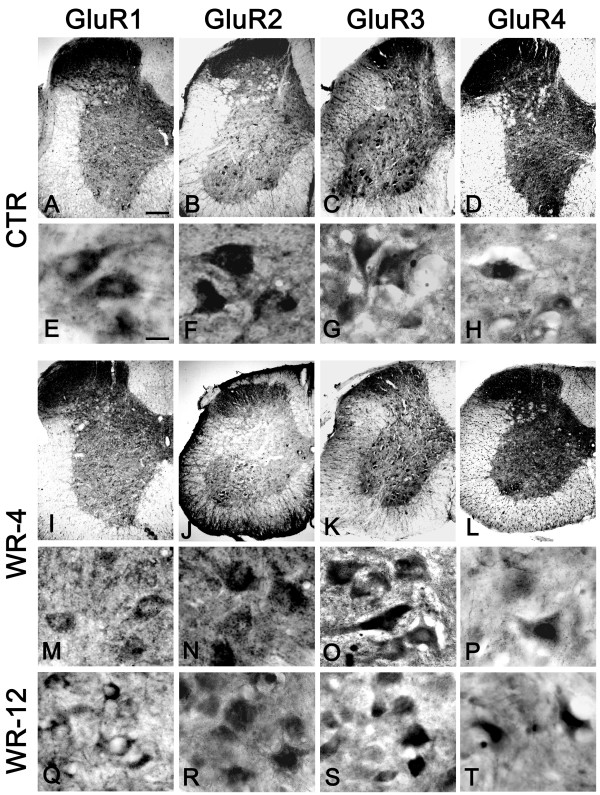
**AMPA receptor subunits in the spinal cord of control and wobbler mice**. Representative photomicrographs showing the pattern of AMPA immunostaining in the cervical spinal cord of four-week-old healthy mice (A-D) and age-matched wobbler mice (I-L). Subcellular localisation of the different AMPA receptor subunits in four-week-old healthy mice is shown in panels (E-H) Panels (M-P) show the pattern of staining in motoneurons of the cervical spinal cord from four-week-old wobbler mice. The distribution of GluR 1–4 in the cervical spinal cord of 12-week-old wobbler mice is shown in panels (Q, R, S, T). Scale bar, A-D, I-L 100 μm. E-H, M-T 20 μm.

The patterns of staining for GluR1 in the whole cervical spinal cord of four-week-old wobbler mice and healthy littermates e shown in figure 1, panels A, I. In both sections there was marked immunoreactivity in the dorsal horns and weak but homogenous staining in whole gray matter; the white matter was almost unstained for GluR1. No real differences were seen between wobbler and healthy mice sections.

Figure 1, panels E, M, shows the distribution and localization of GluR1 staining in motoneurons in the cervical spinal cord of healthy mice (E) and early symptomatic wobbler mice (M). The distribution of GluR1 in motoneurons of healthy mice was detectable only outside the nucleus possibly on the cell surface and in the cytoplasm. Motoneurons from the cervical region of wobbler mice showed weaker staining and a diffuse immunoreactivity not selective to neurons, colocalisation experiments have demonstrated to be related to glial cells (figure 3C–F); this novel pattern of GluR1 staining was also evident in motoneurons of 12-week-old wobbler mice (Q).

##### GluR2

GluR2 immunoreactivity in the cervical spinal cord of four-week-old wobbler mice and healthy littermates is shown in figure 1, panels B, J. GluR2 showed an intense staining in the whole gray matter, mainly in the dorsal horns and in neurons of ventral horns. In the anterior horn, large neurons in the lamina IX showed intense immunoreactivity. No marked differences were seen between healthy mice (B) and wobbler mice (J).

The distribution and localization of GluR2 immunostaining in anterior horn neurons of the cervical spinal cord in four-week-old wobbler mice and healthy littermates is shown in figure 1, panels F, N. Motoneurons were intensely stained in wobbler mice (N) and healthy littermates (F). The same pattern of staining for GluR2 was seen in surviving motoneurons in 12-week-old wobbler mice (R).

##### GluR3

Cervical spinal cord sections showed strong immunostaining for GluR3 in the dorsal horns, both in wobbler and healthy littermates (figure 1 panels C, K). The anterior horn showed spotty staining, mainly in the central and lateral columns of lamina IX motoneurons. Observation of the anterior horn at higher magnification clearly confirmed that this strong immunoreactivity for GluR3 was related to large neurons.

As shown in figure 1, panels G and O, GluR3 was markedly expressed in the whole surface of the cell body in motoneurons of the cervical spinal cord and also in the proximal region of its prolongations, likely axons. There was no marked difference in localization, distribution and density of staining for GluR3 in motoneurons between wobbler mice (O) and healthy littermates (G) in either the presymptomatic or late symptomatic stage (S).

##### GluR4

Staining for GluR4 was mainly localized in the dorsal horns. In the cervical region of four-week-old wobbler mice modest but detectable immunostaining was also observed in the anterior horns, but in fibers more than in cell bodies (figure 1 panels D, L). Observation of anterior horn neurons at higher magnification clearly revealed a marked immunoreactivity around the nuclear area and a weaker signal in the periphery in unaffected (figure 1H), in early (P) and late symptomatic mice (T).

#### NR1 and NR2A

**Figure 2:**
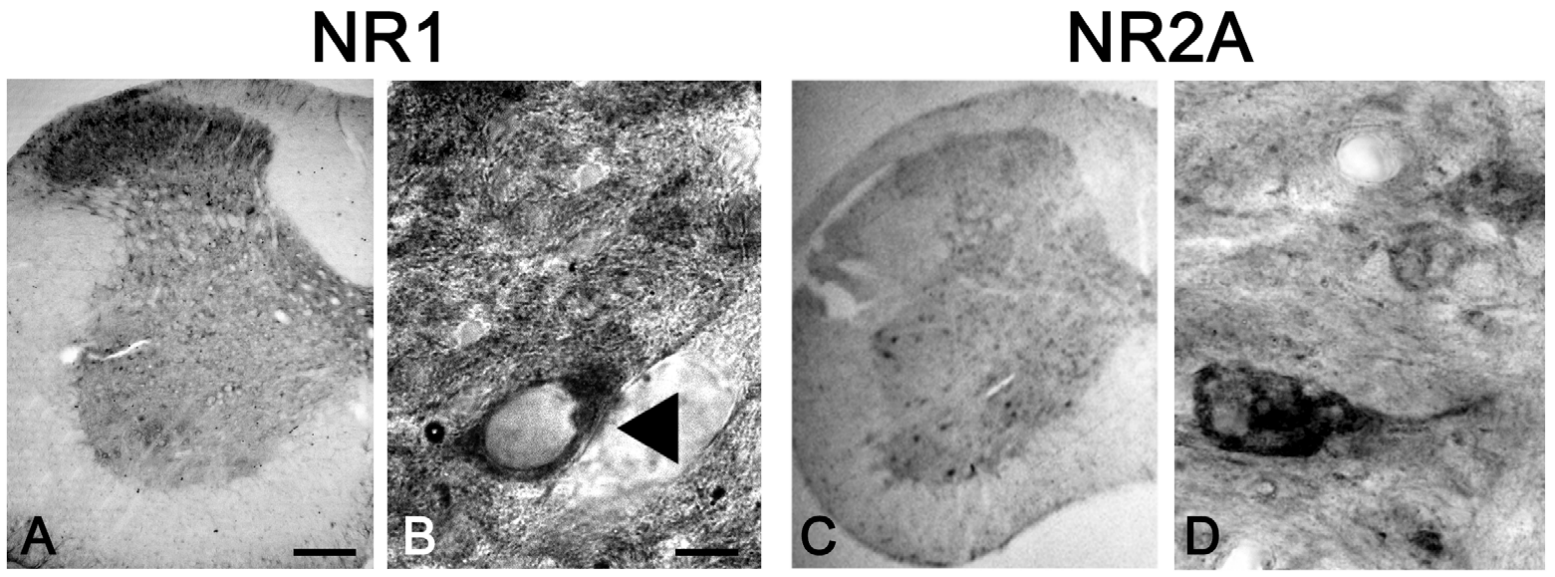
**NMDA receptor subunits in spinal cord of wobbler mice**. Representative photomicrographs showing NR1 (A,C) and NR2A (B,D) immunostaining in the cervical spinal cord of four-week-old wobbler mice (B). Scale bar, A, B 100 μm. C, D 20 μm.

Figure 2 shows the pattern of staining for two NMDA receptor subunits (NR1 and NR2A) in the spinal cord of early symptomatic wobbler mice. In wobbler mice and healthy littermates (not shown) the staining for NR1 and NR2A was weaker and more homogeneously diffused in the whole grey matter than for AMPA receptor subunits. The antibody directed against the NR1 subuni showed a marked immunoreactivity mainly in dorsal horns and in neurons localized in the lamina X close to the central canal. In the ventral horns the staining was very weak and in the lamina IX large- neurons, presumably motoneurons, shoed pattern of immunoreactivity confined to the extreme periphery of the cell, likely delimiting the plasma membrane. No differences between affected mice and healthy littermates have been observed either in the distribution of these subunit in the different areas of spinal cord or in the specific motoneuron staining.

**Figure 3:**
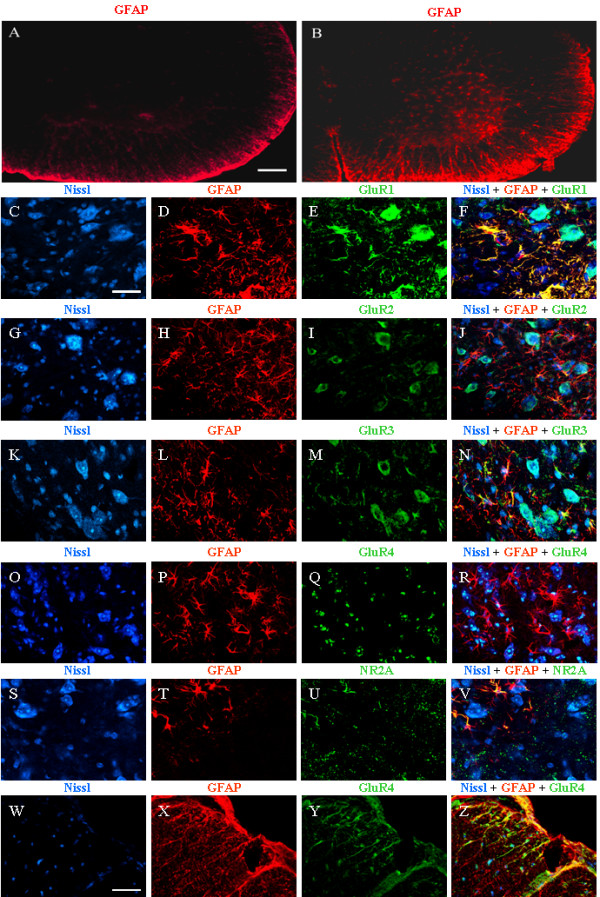
**ionotropic glutamate receptors in the lamina IX in the cervical spinal spinal cord of four-week-old wobbler mice**. UPPER PANELS. Representative photomicrographs showing the patterns of GFAP immunostaining in the cervical spinal cord region of 12-week-old mice (A) and age-matched symptomatic wobbler mice (B). CENTRAL PANELS (C-V). Triple staining experiments using Nissl (C, G, K, O, S, purple colour) GFAP (D, H, L, P, T, red colour) and GluR 1–4 or NR2A (E, I, M, Q, U, green colour) in the anterior horn of four-week-old wobbler mice. The merge between the three different colours is shown in panels F, J, N, R, V. LOWER PANELS (W-Z). Triple staining showing Nissl (W), GFAP (X), GluR4 (Y) and the merge between the three different colurs (Z), in the white matter in the anterior region of the cervical spinal cord, four-week-old wobbler mouse. Scale bar, A-B, 100 μm. C-V, 30 μm. W-Z 40 μm.

Different to NR1 immunostaining the immunoreactivity of NR2A was nearly absent in dorsal and central areas and was mainly concentrated in ventral neurons (figure 2C). In motoneurons the immunoreactivity for NR2A was detectable in the surface corresponding to the cell body and in its arborizations (figure 2D); this pattern of staining was similar in healthy mice and in motoneurons of late symptomatic wobbler mice which are not yet degenerated.

#### Lumbar region

Immunohistochemistry experiments for AMPA and MNDA receptor subunits in the lumbar tract of 4- and 12-week-old wobbler and control mice showed the same patterns of staining as in the cervical (data not shown). No difference between unaffected and diseased mice were found in this region.

### Colocalisation experiments

GFAP immunostaining in the cervical region of 12-week-old healthy mice (figure 3A) and in age-matched wobbler mice (figure 3B) confirms that reactive gliosis was exclusively present in affected mice. GFAP positive cells were mainly localized in the anterior horn, close to the injured area. This difference was already detectable in early symptomatic wobbler mice (figure 3, panels D,H,L,P,T).

Figure 3, panels C-V, show triple staining experiments of confocal microscopy in the lamina IX of the cervical spinal cord of four-week-old wobbler mice using fluorescent Nissl tracer to stain neurons (first column, purple), anti-GFAP antibody to stain astrocytes (second column, red) and specific antibodies to AMPA receptor subunits and NR2A (third column, green). The same experiment was carried out also in healthy mice (not shown) but the lack of immunoreactivity for GFAP did not give us any significant information about the glial localisation of such subunits in unaffected mice.

Figure 3F shows the merge between Nissl staining (C), GFAP immunoreactivity (D) and GluR1 expression (E) in the lamina IX in the cervical region of four-week-old wobbler mice. This experiment confirmed that GluR1 immunoreactivity was abundant in large motoneurons, with a lower profile of expression in smaller Nissl-positive cells and strong immunoreactivity in GFAP-positive cells.

The merge between Nissl staining (G), GFAP immunoreactivity (H) and GluR2 expression (I) is shown in figure 3J. GluR2 was almost exclusively expressed in Nissl-positive cells, either in large or in small neurons, while colocalisation between GFAP and this subunit was very weak and spare.

Figure 3, panels K-N, shows the staining for Nissl (K), GFAP (L), GluR3 (M) and the merge among the three colours (N). GluR3 was abundantly expressed in almost all neurons and also colocalised with GFAP-positive cells.

GluR4 immunoreactivity, figure 3Q, was very low and confined to large Nissl-positive neurons (O, R), whereas GFAP-positive cells (P) showed no colocalisation with this subunit (R). Interestingly, colocalisation experiments in the white matter of anterior horn in the same section, figure 3, panels W-Z, showed a clear pattern of colocalisation (Z) between GFAP-positive radial glia (X) and GluR4 (Y).

Figure 3, panels S-V, shows the expression of NR2A (U), Nissl (S), GFAP (T) and the merge among the three images (V). Although NR2A seemed to show a lower staining in neurons compared to single immunohistochemical experiments (figure 2C,D) there was a clear colocalisation between this subunit and GFAP-positive cells (figure 3V).

To clarify the subcellular distribution of AMPA receptor subunits, double staining experiments were done in the cervical region of four-week-old wobbler mice and healthy littermates.

**Figure 4:**
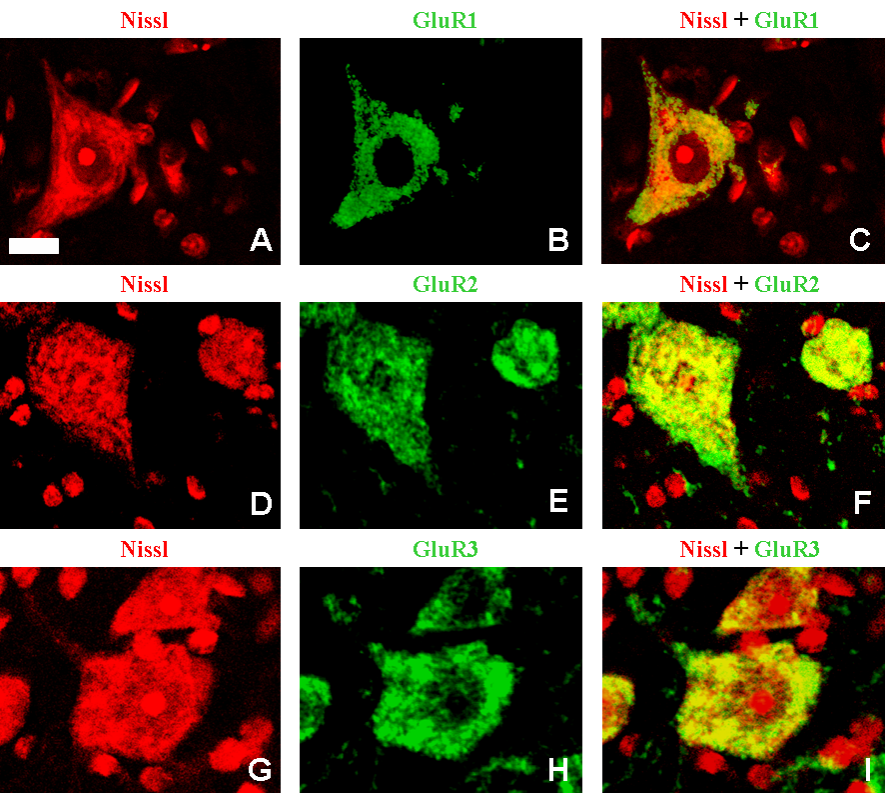
**cellular localization of GluR 1–3 subunits in motneurons in the cervical spinal spinal cord of four-week-old wobbler mice**. LEFT COLUMN (A, D, G). Panels A, D, G, show the profile of Nissl staining (red) in large neurons localized in the lamina IX of the cervical spinal cord of four-week-old wobbler mouse. CENTRAL COLUMN (B, E, H). Central panels show the staining for the three different AMPA receptor subunits, GluR1 (B), GluR2 (E), GluR3 (H) (green) in lamina IX of the cervical spinal cord of four-week-old wobbler mouse. RIGHT COLUMN (C, F, I). Panels C, F, I show the localisation of GluR1 (C), GluR2 (F) and GluR3 (I) in Nissl-positive large neurons of the lamina IX. Jellow staining is the area of merge between Nissl and AMPA receptor subunits. Scale bar, 15 μm.

Figure 4, panels A, D, G, shows high magnification pictures of Nissl-positive large neurons localized in lamina IX, presumably motoneurons. Due to the abundance of cytoplasmic ribonucleic acids in neurons Nissl staining was not only a reliable marker to recognize neurons but also to selectively stain the cytoplasm. Therefore the merge between GluR1 (B), GluR2 (E) or GluR3 (H) and Nissl-positive neurons allowed to show the expression of AMPA receptor subunit in close proximity to the axons and dendrites. The merge between Nissl and each single AMPA receptor subunit, figure 4, panels C, F, I, shows that the immunoreactivity for GluR1 (C), GluR2 (F) and GluR3 (I) was not only confined to perykarion but it was also evident in the proximal part of its arborizations morphologically similar to axon hillock.

### Western blotting analysis

**Figure 5:**
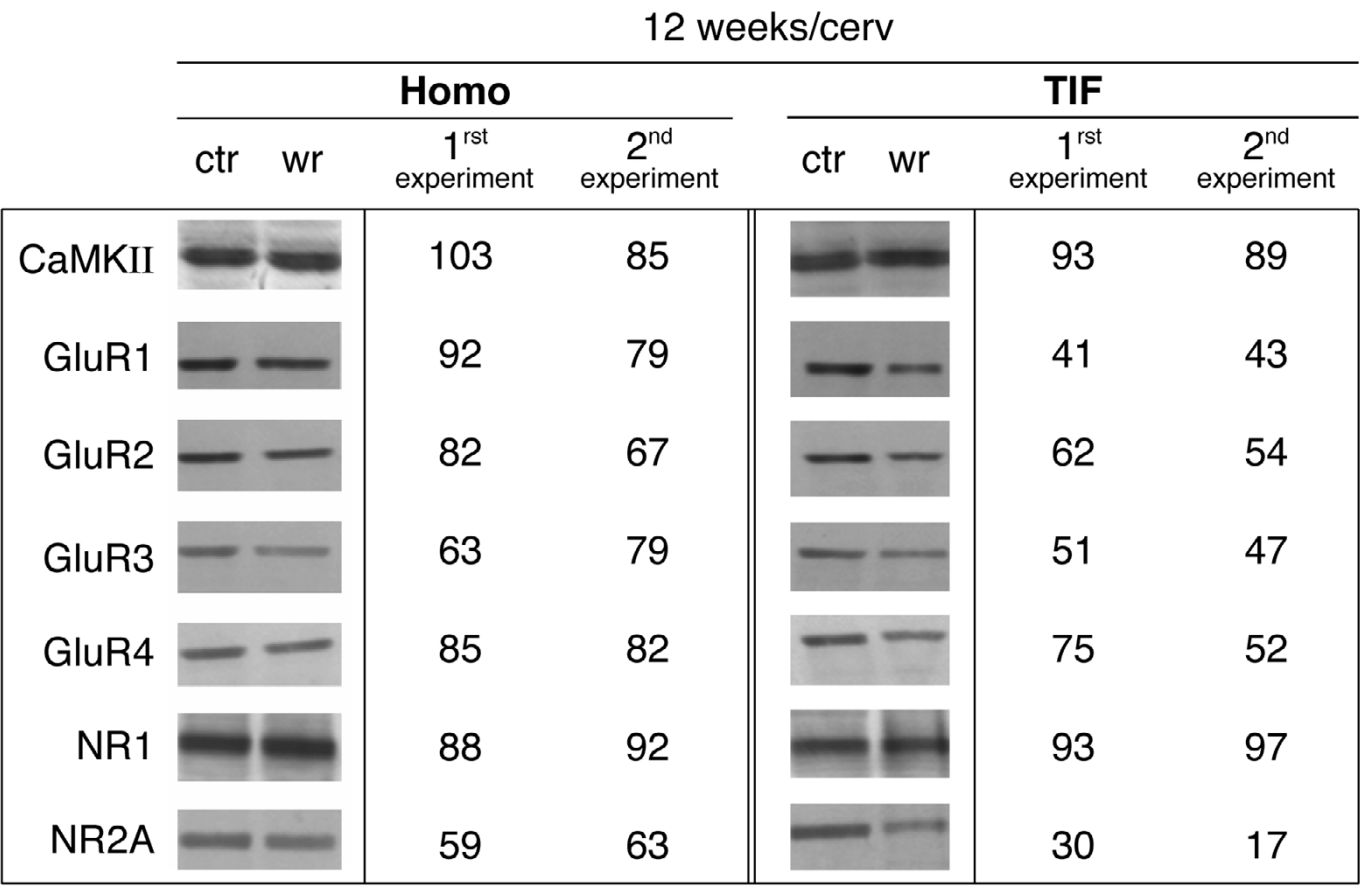
**western blotting analysis and quantification of the percentage of AMPA and NMDA receptor subunits expression in spinal cord of wobbler mice**. Representative immunoblot from cervical spinal cord samples from 12-week-old healthy mice and age-matched wobbler mice. Left column: whole spinal cord homogenates. Right column: spinal cord TIF. Quantification of the mean values of immunodensity for CaMK, AMPA and NMDA receptor subunits, obtained from the cervical spinal wobbler mice in whole spinal cord homogenates (Homo) and in TIF. Data are representative of two independent experiments on two different homogenate preparations of four pooled animals for each group and replicated two times in each homogenate preparation. Values reported represent the percentage of the mean values of immunodensity obtained in 12-week-old wobbler mice compared to the levels measured in healthy littermates and normalized to 100. Both preparations samples were analyzed by Western blot analysis with CaMKII, GluR1, GluR2, GluR3, GluR4, NR1, and NR2A antibodies. The same amount of protein was loaded per lane.

AMPA and NMDA receptor subunit protein levels were examined by western blot analysis in whole homogenate and TIF prepared from cervical and lumbar spinal cord of 4- and 12-week-old wobbler mice and healthy littermates. Protein yields were similar in TIF from all groups and the same amount of whole homogenate and TIF proteins was applied to SDS-gel and electroblotted. At both stages of the disease no differences between wobbler mice and healthy littermates were found in the levels of expression of any proteins in the homogenate fractions, in either lumbar or cervical spinal cord region (figure 5). Alterations in the protein levels in the TIF were observed in late symptomatic wobbler mice aged 12 weeks (figure 5), but not in early symptomatic mice.

The quantification of the optical densities of proteins in whole homogenate fraction and TIF, performed in two different experiments from the cervical region of 12-week-old wobbler mice, is shown in figure 5. Mean values from wobbler mice were expressed as percentage of the mean values obtained from healthy littermates.

Although statistical comparison cannot be performed, the quantification showed that there was a strong reduction for NR2A and GluR1 in the TIF, but not in whole homogenate. In TIF of 12-week-old wobbler mice GluR2 and GluR3 were reduced to a lower extent.

In the lumbar spinal cord there was no change of glutamate receptor subunits for the TIF compartment even though a surprising increase in NR2A was detected in lumbar region of four-week-old of wobbler mice.

GFAP levels in whole homogenates of cervical spinal cord in either 4- and 12-week-old wobbler mice are markedly increased [17], however no GFAP expression in TIF samples was found from cervical and lumbar spinal cord of wobbler mice and healthy littermates at four and 12 weeks of age (data not shown).

## Discussion

NR2A and GluR1 receptor subunits in TIF samples from the cervical spinal of cord wobbler mice decreased only in the late stage of the disease, when the two thirds of motoneurons were lost, but not in the early stage. This is an important point of discussion because between the fourth and fifth weeks of life the rate of motoneuron death peaks in the cervical region of wobbler mice (25% of total motoneurons disappears), but no simultaneous alteration in the expression of AMPA and NMDA receptors in post-synaptic densities are present. The lack of lowering of AMPA and NMDA receptor subunits levels in TIF from early symtpomatic wobbler mice, when at least 25% of motoneurons are lost, might be to due to the fact that post-synaptic densities are not only related to motoneurons, and our immunohistochemical experiments clearly showed that AMPA and NMDA receptor subunits are expressed in almost all neuronal populations, particularly the dorsal horn neurons.

In addition, immunohistochemical studies indicated that the localisation and the distribution of AMPA and NMDA receptor subunits in the whole cervical region of both early and late symptomatic wobbler mice was no different from that of healthy littermates. It is of interest that the decrease of AMPA and NMDA receptor in TIF was not found in the lumbar spinal cord region, in either early or late stages of the disease, where motoneurons were not lost. These observations suggest that the decrease in GluR1 and NR2A receptor subunits in TIF from the cervical spinal cord of late symptomatic wobbler mice might be a consequence of motoneuron loss. Differently from the TIF, the whole homogenate fraction from cervical region of 12-week-old wobbler mice and healthy littermates contained the same amounts of AMPA and NMDA receptor subunits. However, TIF includes glutamate receptors expressed in the post-synaptic densities of spinal neurons, and the glial component was nearly absent in this preparation [20,21]. Our study using triple-immunofluorescence experiments showed that GluR1 and NR2A were highly expressed in activated astrocytes, and marked reactive gliosis selectively occurs in the cervical spinal cord of wobbler mice during the progression of the disease [17]. Thus, in the whole homogenate from affected region, the expression of GluR1 and NR2A receptors in activated astrocytes may partially mask the difference of these subunits found in TIF.

Although in the cervical region of wobbler mouse there is no evidence of increased glutamate release from the corticospinal tract, a defective mechanism regulating glutamate/glutamine synthesis and/or influx or efflux in purified cultures of astrocytes from symptomatic wobbler mice has been reported [22]. Our study evidenced that astrocytes in the cervical region of wobbler mice lack the Ca^++ ^impermeable AMPA receptor subunits (GluR2) and suggests an interesting scenario about neurons-glia crosstalk in wobbler motoneuron disease. Further investigation of the susceptibility to excitotoxic agents in motoneurons from the cervical spinal cord of wobbler mice would help to characterize the involvement of glutamate-induced excitotoxicity in this model.

A less direct but nevertheless reliable approach to study the different sensitivity of AMPA receptors in wobbler mice is chronic treatment with AMPA antagonists.

Their protective role has been reported in different models of motoneuron degeneration. The non-competitive AMPA antagonist ZK187638 significantly reduced the symptoms of neuromuscular deficit and improved the motor behavioural impairment in both SOD1^G93A ^and *mnd *mice, and extended the survival of SOD1^G93A ^transgenic mice [23]. In these latter, the competitive AMPA antagonist RPR119990 significantly improved muscle strength and prolonged survival [24]. We did not find beneficial effects after chronic treatment of wobbler mice with RPR119990 on the progression of the disease, the rate of motoneuron loss and biceps atrophy [25].

Regarding the role of NMDA receptor-mediated injury in wobbler mice, it has been reported that treatment with the non-competitive NMDA receptor antagonist +-5-methyl-10,11-dihydro-5H-dibenzo(a,d)cyclopheten-5,10-imine maleate (MK801), did not delay the progression of the disease [14]. Our characterization seems to be in accordance with this result, suggesting that altered expression of NMDA receptor subunits is not involved in the early symptomatic phase of the disease.

Although further experiments with other AMPA and NMDA receptor antagonists are required to confirm our results, this pharmacological evidence adds relevance to the results reported in the present study and makes even more convincing that no alteration in glutamate receptors is involved in the wobbler mouse motoneuron disease.

## Conclusion

The main evidence from this study is that the decrease in the amount of GluR1 and NR2A receptor subunits in TIF in late symptomatic wobbler mice is not related to an early reduction in the expression of these subunits in degenerating motoneurons, but more likely is related to the reduction in motoneuron number at the late stage of the disease.

## Methods

### Animals

Wobbler mice and homozygous healthy littermates (NFR/wr strain, NIH, Animal Resources, Bethesda, USA) were bred at Charles River Italia (Calco, Lecco, Italy). Mice were maintained at 21 ± 1°C with relative humidity 55 ± 10% and a 12-hour light/dark cycle. Food (standard pellets) and water were available ad libitum.

Procedures involving animals and their care were conducted in conformity with the institutional guidelines that are in compliance with national (D.L. n° 116, G.U. suppl. 40, 18 Febbraio 1992, circolare n° 8, G.U. 14 Luglio 1994) and international laws and policies (EEC Council Directive 86/609, OJ L 358, 1, December 12, 1987; NIH Guide for the Care and Use of Laboratory Animals, US National Research Council 1996).

### Experimental animals

In order to identify wr/wr homozigous mice, healthy homozygous +/+ and healthy heterozygous wr/+ littermates, a genotyping analysis was performed. An Alu I restriction polymorphism of a Cct 4 amplification product was used for testing the allelic status at the wr locus (Rathke-Hartlieb 1999).

Early ymptomatic wobbler mice can be easily recognized by their phenotypical features. From 3^rd ^week of age, wobbler mice begin to growth slower than healthy littermates and, only a week later, they are 40–50% (also depending on the strain) smaller that their age-matched healthy mice. At the 3^rd^–4^th ^week wobbler mice already show an altered position of fingers, wrists and paws. This alteration derives from muscular atrophy and produces unsteady gait with a discrete tremor. Afterwards, instability and wobbling of the gait develop progressively thus producing alteration in walking that is a typical feature of motor impairment occurring in the wobbler mice. As previously proposed by Kozachuk and colleagues, these two parameters of abnormalities can be useful to determine the degree of clinical worsening during this phase of the disease [26].

### Immunohistochemistry

To ensure optimal quality of spinal cord tissues for histochemical determinations, tissues were fixed following the transcardial perfusion method using a solution of 4% paraformaldehyde (w/v) in 0.1 M phosphate buffered saline (PBS) (pH 7.4). After perfusion, the isolated backbone was postfixed for 3 hours at 4°C in the same solution. After postfixation the spinal cord was gently removed from the vertebral column and cryoprotected by three serial 2-h incubations at 4°C, in 0.1 M PBS, containing increasing concentrations of sucrose (10%, 20% and 30%), then dipped in cooled isopentane (-35°C to -45°C) to quickly freeze them.

For free-floating immunohistochemistry, micrometric sections (30 μm thick) were placed in plate wells containing PBS, then rinsed three times (10 min each) to remove the Tissue-Tek^® ^O.C.T.™ solution used to surround and cover spinal tissues and ensure optimal cutting.

Before incubation with specific antibodies directed against the GluR-1, 2, 4 subunits, the same experimental procedure was used. After three rinses in 0.1 M PBS, sections were preincubated in PBS containing 5% foetal bovine serum and then incubated in PBS containing 0.5% Triton-X100 for 1 hour at room temperature. For GluR3 immunostaining, preincubation in PBS containing 0.5% albumin for 24 hours at 4°C was done to reduce non-specific staining.

For all AMPA receptor subunits, sections were incubated overnight at 4°C in a solution of PBS containing 0.1% Triton-X100 and 3% FBS with the specific antibodies: anti-rabbit polyclonal antibody raised against GluR-1 (AB1504, Chemicon International, Temecula, CA, US; 1:200); anti-mouse monoclonal antibody raised against GluR-2 (MAB397, Chemicon International, Temecula, CA, US; 1:1000); anti-goat polyclonal antibody raised against GluR-3 (sc-7613, Santa Cruz Biotechnology, Santa Cruz, CA, US; 1:400) and anti-rabbit polyclonal antibody raised against GluR-4 (AB1508, Chemicon International, Temecula, CA, US; 1:50). For the NMDA receptor we used an anti-mouse monoclonal NR1 antibody (Catatolg No 32-0500, Zymed, Invitrogen, Carlsbad, CA, US; 1:100) and an anti-rabbit polyclonal NR2A antibody (Molecular Probes, Invitrogen, Carlsbad, CA, US; 1:100).

After incubation with the primary antibody and three rinses in PBS at room temperature, all the sections were incubated for 2 hours at room temperature in PBS containing 1% FBS and the appropriate secondary antibody (1:100).

For each AMPA and NMDA receptor subunit, after incubation with the secondary antibody the sections were rinsed three times (5 min) then incubated for 1 hour in a solution containing 1% avidin and biotinylated horseradish peroxidase (ABC kit) in PBS 0.1 M, pH 7.4. After one rinse in PBS and two rinses in Tris buffered saline (TBS) 0.1 M, pH 7.8–8.3, the sections were incubated for a few minutes in TBS containing 0.5% diamminobenzidine (DAB) (w/v) and H_2_O_2 _(0.6 μL for 1 mL of solution, was added just before application). As DAB is a photosensitive molecule, it was dissolved and stored in a dark vial until incubation.

In order to avoid possible false positive results control experiments were done using the primary or the secondary antibodies alone. All experiments did not produce staining in tissues examined.

### Immunofluorescence

For colocalisation experiments to characterize AMPA and NR2A receptor subunit expression in astrocytes and neurons in the cervical spinal cord of four-week-old wobbler mice and healthy littermates perfused samples were sectioned at a thickness of 15 μm. As either anti-GFAP antibody or anti-NR1 antibody are monoclonal anti-mouse we could not perform colocalisation experiments among these two proteins. For immunofluorescence experiments the following antibodies were used: (rabbit anti-GluR1 polyclonal antibody AB-10129, Immunological Sciences, Roma, IT, 1:200), (rabbit anti-GluR2 polyclonal antibody AB-10699, Immunological Sciences, Roma, IT, 1:200), (goat anti-GluR3 polyclonal antibody sc-7613, Santa Cruz Biotechnology, Santa Cruz, CA, US; 1:400) (rabbit anti-GluR4 polyclonal antibody AB-10122, Immunological Sciences, Roma, IT, 1:50), (rabbit anti-NR2A polyclonal antibody AB-10675, Immunological Sciences, Roma, IT, 1:200). All antibodies were incubated as reported in the immunohistochemical methods section. Secondary antibodies (Alexa-488, Molecular Probes, 1:1000) were incubated for 2 h at room temperature. All sections were incubated using a specific marker for astrocytes (anti-mouse monoclonal GFAP antibody, MAB-12029 Immunological Sciences, Roma, IT, 1:5000) at a dilution of. The secondary antibody (Alexa-Cy5 conjugated, Molecular Probes) was used to visualize astrocyte staining. For Nissl immunofluorescence, sections were incubated 30 min with 530–615 NeuroTrace Fluorescent Nissl reagent (Molecular Probes, 1:100) at room temperature.

Sections were observed with an *Olympus Fluoview *microscope BX61 with confocal system FV500. Images were pseudocolored (red for the GFAP-associated staining and green for the AMPA and NMDA receptor associated staining, purple for Nissl-associated staining) and the signal obtained from the three different channels was automatically merged by Olympus fluoview software.

### Western blot analysis

Subcellular fractionation of spinal cord tissue was done as previously reported, with minor modifications [21]. Two different preparations, whole homogenate and TIF, were obtained using four pooled animals for each group. Cervical and lumbar spinal cord were homogenized in ice-cold 0.32 M sucrose containing 1 mM Hepes, 1 mM MgCl_2_, 1 mM EDTA, 1 mM NaHCO_3_, 0.1 mM PMSF, pH 7.4, with complete sets of protease inhibitors (Complete™, Roche Diagnostics, Basel, Switzerland) and phosphatase inhibitors (Sigma-Aldrich; Saint Louis, MO, US). The homogenized tissue was centrifuged at 1,000 × g for 10 min. The supernatant (S1) was centrifuged at 13,000 × g for 15 min to obtain a crude membrane fraction (P2 fraction). The pellet was re-suspended in 1 mM Hepes + Complete™ in a glass-glass Potter apparatus and centrifuged at 100,000 × g for 1 h. The pellet (P3) was resuspended in buffer containing 75 mM KCl and 1% Triton-X100 and centrifuged at 100,000 × g for 1 h. The final pellet (P4) was homogenized in a glass-glass Potter apparatus in 20 mM Hepes. Then an equal volume of glycerol was added and this fraction, referred to as TIF, was stored at -80°C until processing. TIF was used instead of the classical post-synaptic density (PSD) because the amount of starting material was very limited. The protein composition of this preparation was, however, carefully tested for the absence of presynaptic markers (e.g. synaptophysin) [21]. Nitrocellulose papers were blocked with 10% albumin in TBS, and incubated for 2 h at room temperature with the primary antibodies NR1 (diluted 1:1000), NR2A (1:1000), GluR1 (1:2000), GluR2 (1:1000), GluR3 (1:2000), GluR4 (1:2000), alphaCaMKII (Chemicon International, Temecula, CA, US; 1:3000) in TBS containing 3% albumin. To avoid any difference of interpretation from the results of immunohistochemical experiments and western blot analysis the same batches for AMPA and NMDA receptor antibodies were used for both. After extensive rinsing in 0.1% TBS/Tween, the nitrocellulose papers were incubated with horseradish peroxidase-conjugated secondary antibodies [goat anti-rabbit, for polyclonal antibodies, dilution 1:10000 (Pierce Biotechnology Inc., Rockford, IL, U.S.); goat anti-mouse, for monoclonal antibodies, dilution 1:20000 (Pierce Biotechnology Inc., Rockford, IL, U.S.)] and the antigen-antibody complex was revealed by enhanced chemiluminescence (ECL; Amersham International, Little Chalfont, Buckinghamshire, UK).

### Densitometric measurement

Optical density was quantified using the AIS image analyzer (Imaging Research Inc., Ontario, Canada).

## Authors' contributions

T.M., M.DiL. and M.M.C. conceived the project. P.B., S.B. and E.F. did the immunohistochemical studies. A.C genotyped the animals. F.G and A.L. ran the biochemical studies.

All authors read and approved the final manuscript.

## References

[B1] Brooks BR (1994). El Escorial World Federation of Neurology criteria for the diagnosis of amyotrophic lateral sclerosis. Subcommittee on Motor Neuron Diseases/Amyotrophic Lateral Sclerosis of the World Federation of Neurology Research Group on Neuromuscular Diseases and the El Escorial "Clinical limits of amyotrophic lateral sclerosis" workshop contributors. J Neurol Sci.

[B2] Rowland LP (1998). Diagnosis of amyotrophic lateral sclerosis. J Neurol Sci.

[B3] Cudkowicz ME, McKenna-Yasek D, Sapp PE, Chin W, Geller B, Hayden DL, Schoenfeld DA, Hosler BA, Horvitz HR, Brown RH (1997). Epidemiology of mutations in superoxide dismutase in amyotrophic lateral sclerosis. Ann Neurol.

[B4] Mennini T, Bendotti C, Ferrarese C and Beal MF (2004). Excitotoxicity in amyotrophic lateral sclerosis: selective vulnerability of motor neurons. Excitotoxicity in neurological diseases New Therapeutic Challenge.

[B5] Rothstein JD, Jin L, Dykes-Hoberg M, Kuncl RW (1993). Chronic inhibition of glutamate uptake produces a model of slow neurotoxicity. Proc Natl Acad Sci U S A.

[B6] Raman IM, Zhang S, Trussell LO (1994). Pathway-specific variants of AMPA receptors and their contribution to neuronal signaling. J Neurosci.

[B7] Kawahara Y, Kwak S, Sun H, Ito K, Hashida H, Aizawa H, Jeong SY, Kanazawa I (2003). Human spinal motoneurons express low relative abundance of GluR2 mRNA: an implication for excitotoxicity in ALS. J Neurochem.

[B8] Takuma H, Kwak S, Yoshizawa T, Kanazawa I (1999). Reduction of GluR2 RNA editing, a molecular change that increases calcium influx through AMPA receptors, selective in the spinal ventral gray of patients with amyotrophic lateral sclerosis. Ann Neurol.

[B9] Kuner R, Groom AJ, Muller G, Kornau HC, Stefovska V, Bresink I, Hartmann B, Tschauner K, Waibel S, Ludolph AC, Ikonomidou C, Seeburg PH, Turski L (2005). Mechanisms of disease: motoneuron disease aggravated by transgenic expression of a functionally modified AMPA receptor subunit. Ann N Y Acad Sci.

[B10] Falconer DS (1956). Wobbler mouse. News Letters.

[B11] Schmitt-John T, Drepper C, Mussmann A, Hahn P, Kuhlmann M, Thiel C, Hafner M, Lengeling A, Heimann P, Jones JM, Meisler MH, Jockusch H (2005). Mutation of Vps54 causes motor neuron disease and defective spermiogenesis in the wobbler mouse. Nat Genet.

[B12] Mitsumoto H, Ikeda K, Holmlund T, Greene T, Cedarbaum JM, Wong V, Lindsay RM (1994). The effects of ciliary neurotrophic factor on motor dysfunction in wobbler mouse motor neuron disease. Ann Neurol.

[B13] Tsuzaka K, Ishiyama T, Pioro EP, Mitsumoto H (2001). Role of brain-derived neurotrophic factor in wobbler mouse motor neuron disease. Muscle Nerve.

[B14] Krieger C, Perry TL, Hansen S, Mitsumoto H, Honore T (1992). Excitatory amino acid receptor antagonist in murine motoneuron disease (the wobbler mouse). Can J Neurol Sci.

[B15] Gonzalez Deniselle MC, Gonzalez SL, Lima AE, Wilkin G, De Nicola AF (1999). The 21-aminosteroid U-74389F attenuates hyperexpression of GAP-43 and NADPH-diaphorase in the spinal cord of wobbler mouse, a model for amyotrophic lateral sclerosis. Neurochem Res.

[B16] Blondet B, Barlovatz-Meimon G, Festoff BW, Soria C, Soria J, Rieger F, Hantai D (1992). Plasminogen activators in the neuromuscular system of the wobbler mutant mouse. Brain Res.

[B17] Bigini P, Bastone A, Mennini T (2001). Glutamate transporters in the spinal cord of the wobbler mouse. Neuroreport.

[B18] Krieger C, Wagey R, Shaw C (1993). Amyotrophic lateral sclerosis: quantitative autoradiography of [3H]MK-801/NMDA binding sites in spinal cord. Neurosci Lett.

[B19] Tomiyama M, Kannari K, Nunomura J, Oyama Y, Takebe K, Matsunaga M (1994). Quantitative autoradiographic distribution of glutamate receptors in the cervical segment of the spinal cord of the wobbler mouse. Brain Res.

[B20] Gardoni F, Schrama LH, Kamal A, Gispen WH, Cattabeni F, Di Luca M (2001). Hippocampal synaptic plasticity involves competition between Ca2+/calmodulin-dependent protein kinase II and postsynaptic density 95 for binding to the NR2A subunit of the NMDA receptor. J Neurosci.

[B21] Finardi A, Gardoni F, Bassanini S, Lasio G, Cossu M, Tassi L, Caccia C, Taroni F, LoRusso G, Di Luca M, Battaglia G (2006). NMDA receptor composition differs among anatomically diverse malformations of cortical development. J Neuropathol Exp Neurol.

[B22] Hantaz-Ambroise D, Cambier D, Ait-Ikhlef A, Parvy P, Murawsky M, Rieger F (1995). Excess extracellular and low intracellular glutamate in poorly differentiating wobbler astrocytes and astrocyte recovery in glutamine-depleted culture medium. J Neurochem.

[B23] Tortarolo M, Grignaschi G, Calvaresi N, Zennaro E, Spaltro G, Colovic M, Fracasso C, Guiso G, Elger B, Schneider H, Seilheimer B, Caccia S, Bendotti C (2006). Glutamate AMPA receptors change in motor neurons of SOD1G93A transgenic mice and their inhibition by a noncompetitive antagonist ameliorates the progression of amytrophic lateral sclerosis-like disease. J Neurosci Res.

[B24] Canton T, Bohme GA, Boireau A, Bordier F, Mignani S, Jimonet P, Jahn G, Alavijeh M, Stygall J, Roberts S, Brealey C, Vuilhorgne M, Debono MW, Le Guern S, Laville M, Briet D, Roux M, Stutzmann JM, Pratt J (2001). RPR 119990, a novel alpha-amino-3-hydroxy-5-methyl-4-isoxazolepropionic acid antagonist: synthesis, pharmacological properties, and activity in an animal model of amyotrophic lateral sclerosis. J Pharmacol Exp Ther.

[B25] Fumagalli E, Bigini P, Barbera S, De Paola M, Mennini T (2006). Riluzole, unlike the AMPA antagonist RPR119990, reduces motor impairment and partially prevents motoneuron death in the wobbler mouse, a model of neurodegenerative disease. Exp Neurol.

[B26] Kozachuk WE, Mitsumoto H, Salanga VD, Beck GJ, Wilber JF (1987). Thyrotropin-releasing hormone (TRH) in murine motor neuron disease (the wobbler mouse). J Neurol Sci.

